# Comparison of Respiratory Resistance Measurements Made with an Airflow Perturbation Device with Those from Impulse Oscillometry

**DOI:** 10.1155/2013/165782

**Published:** 2013-04-04

**Authors:** J. Pan, A. Saltos, D. Smith, A. Johnson, J. Vossoughi

**Affiliations:** ^1^Fischell Department of Bioengineering, University of Maryland, College Park, MD 20742, USA; ^2^Engineering and Scientific Research Associates, Olney, MD 20832, USA; ^3^School of Medicine, University of Maryland, Baltimore, MD 21201, USA

## Abstract

The airflow perturbation device (APD) has been developed as a portable, easy to use, and a rapid response instrument for measuring respiratory resistance in humans. However, the APD has limited data validating it against the established techniques. This study used a mechanical system to simulate the normal range of human breathing to validate the APD with the clinically accepted impulse oscillometry (IOS) technique. The validation system consisted of a sinusoidal flow generator with ten standardized resistance configurations that were shown to represent a total range of resistances from 0.12 to 0.95 kPa*·*L^−1^ *·*s (1.2–9.7 cm H_2_O*·*L^−1^ *·*s). Impulse oscillometry measurements and APD measurements of the mechanical system were recorded and compared at a constant airflow of 0.15 L*·*s^−1^. Both the IOS and APD measurments were accurate in assessing nominal resistance. In addition, a strong linear relationship was observed between APD measurements and IOS measurements (*R* ^2^ = 0.999). A second series of measurements was made on ten human volunteers with external resistors added in their respiratory flow paths. Once calibrated with the mechanical system, the APD gave respiratory resistance measurements within 5% of IOS measurements. Because of their comparability to IOS measurements, APD measurements are shown to be valid representations of respiratory resistance.

## 1. Introduction

### 1.1. Respiratory Resistance

Respiratory resistance is proportional to the total opposition to breathing caused by frictional forces in the airway passages. At any given time, respiratory resistance is equal to the ratio of respiratory pressure to airflow, given by

(1) $$\begin{array}{c} R_{\text{res}}=\frac{{\Delta P}_{\text{res}}}{\dot{V}}, \end{array}$$

where *R*_res_ is respiratory resistance, Δ*P*_res_ is the respiratory pressure gradient, and $\dot{V}$ is airflow [1, 2].

Increases in respiratory resistance are symptomatic of a number of restrictive and obstructive pulmonary conditions including COPD [3], asthma [4], and bronchiolitis [5]. Useful feedback during administration of endotracheal tubes [6], anesthesia [7], bronchodilator medications [8], and mechanical ventilation [1] can also be given by respiratory resistance measurements.

### 1.2. Measurement Methods

Respiratory resistance is composed of resistances of airways, lung tissue, and chest wall components. Spirometry, specifically peak expiratory flow (PEF) and forced expiratory volume in one second (FEV1), has traditionally been the bedside measurement of choice for diagnosing increased resistance. However, spirometric tests do not directly measure respiratory resistances, are dependent on effort and lung volume, and require complete subject cooperation [9, 10]. Furthermore, since spirometric measurements are principally made during forced exhalation, little insight is normally provided for diagnosing inhalation-related pathologies. Although it has been shown that children as young as 5 years of age are capable of performing spirometry, appropriate coaching is required [11]. 

In contrast to spirometry, passive measurements of total respiratory resistance are a more attractive option for assessing unconscious or noncooperative patients because they require minimal cooperation on the part of the subject. One such method that relies on passive measurements is the forced oscillation technique (FOT). The use of FOT in young and uncooperative patients, for example, has been extensively studied since the 1980s [12], and its theoretical background is comprehensively described.

The FOT determines the mechanical properties of the entire respiratory system (all three components) by superimposing a loudspeaker-generated external pressure signal on the normal breathing pattern of a patient [13]. Impulse oscillometry (IOS) is a form of FOT that uses short duration pressure impulses as the external driver. These impulses are confined to the respiratory system by a parallel bias tube acting as an inertance element to allow patients to breathe regularly to the atmosphere. Dead volume added to the respiratory system by the bias tube can cause respiratory adjustments in airway caliber and depth of breathing. From the in-phase and out-of-phase components of the measured flows and mouth pressures, the IOS machine is able to compute respiratory resistance and reactance over a range of frequencies [14]. IOS has been shown to be effective in measuring lung function both of children [4, 15–18] and of adults [19, 20]. Because it measures total respiratory resistance, IOS is an obvious instrument with which to compare APD measurement

**Figure 1:**
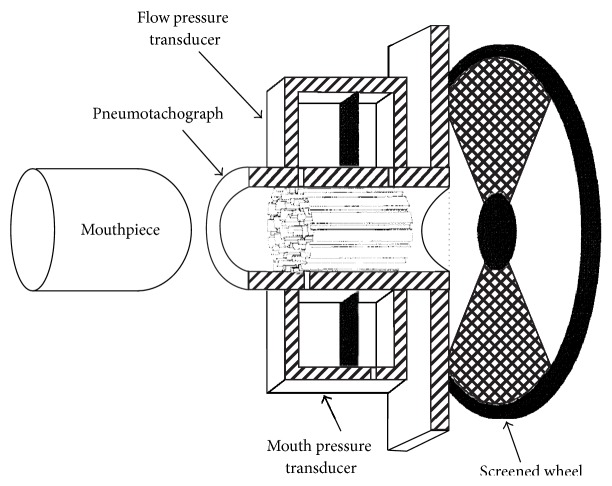
Cut-away diagram of airflow perturbation mechanism. The screened wheel rotates, allowing screened segments to briefly slow, or perturb, airflow passing through the pneumotachograph.

The APD is an instrument that is seeking clinical acceptance. Its advantages over other measurement techniques are that it is small, lightweight, inexpensive, quick, has little external dead volume, and requires little patient cooperation [21–23]. Shown in Figure 1, the APD is composed of a pneumotachograph, two differential pressure transducers, a segmented wheel, motor, and a signal conditioning circuit. The device is held by the patient, while the patient breathes normally through a disposable mouthpiece. A periodic resistance is introduced into the flow path by the rotating wheel with open and screened segments. As the wheel rotates, perturbations are induced in both mouth pressure and flow. The depths of these perturbations depend on relative resistance inside the patient and resistance of the APD itself. Continuous measurement of mouth pressure and flow gives the resistance to the atmosphere of the APD device. From that, it is relatively simple to calculate the patient respiratory resistance as the depth of the pressure perturbation divided by the depth of the flow perturbation. It has been determined from actual measurements that the resistance measured by the APD includes airways, lung tissue, and chest wall components [21–23]. 

The APD is theoretically similar to both the FOT and the interrupter (INT) techniques in measuring respiratory resistance. Like FOT, the APD imposes a periodic perturbation on the breathing waveform. Unlike FOT, the APD determines resistance in the time domain rather than in the frequency domain, easily separates resistance during inhalation from resistance during exhalation, and requires patient breathing to obtain a signal. Some of these characteristics are also shared by INT, but the APD only partially obstructs airflow during tidal breathing. Both pressure and flow signal components are assessed at the same time, so the problem of lung accommodation time that has discredited INT measurements is avoided with the APD. 

 APD measurements have been shown to be reproducible and sensitive to resistance changes in human subjects between the ages of 2 and 88. For example, respiratory resistances measured by the APD clearly show the expected reduction with age as children grow and also the differentiation expected between adult men and women [24]. Similarly, studies have shown the APD to be comparable to the esophageal balloon technique [23] and sensitive to resistance changes in a controlled excised sheep-lung respiratory model [22].

### 1.3. Validation of the APD by Comparing with IOS

The objective of this study was to compare respiratory resistance measurements made with the APD with those made using IOS. This comparison was made using two procedures: (1) in an artificial, nonbiological respiratory model in which resistances were controllable and known and (2) in human volunteers with external resistances added to their respiratory systems. 

There are several important differences between the IOS and APD that influence validation procedures. First, frequency response of the respiratory system is measured by IOS, whereas time response is measured by the APD. Therefore, many measurements are taken by the APD in the time that it takes the IOS machine to take just one. Although the IOS impulse makes the IOS machine capable of quicker readings than the traditional forced oscillation technique, a series of repetitive readings with acceptable coherences is still required for the IOS machine to estimate resistance and reactance. By contrast, the APD is theoretically capable of making measurements as quickly as mouth pressure and airflow can be sampled. To reconcile this difference, IOS and APD measurements can be made more comparable by averaging APD measurements over a discrete duration, such as one minute. In addition, because the IOS impulse contains a range of frequencies, IOS can give resistance and reactance measurements at several different frequencies simultaneously. The commercially available IOS instrument used in the system described in this paper displayed resistance values at 5 Hz and 20 Hz as R5 and R20, respectively. Unless the APD wheel speed is changed, the APD is capable of expressing resistance at only the one wheel speed.

The second important difference is that the APD signal is developed across and through the varying resistance of the wheel. Consequently, there is no phase angle between mouth pressure and flow, so reactance cannot be measured as directly with the APD as with IOS. As a result, the system by which the devices are validated must be dominated by resistance and not reactance. Moreover, since the IOS machine produces its signal with a speaker-like transducer, its signal-to-noise (S/N) ratio is highest with no respiratory flow rate (no noise, only signal). On the other hand, the APD requires a respiratory flow rate in order to produce a signal, so the higher the respiratory flow rate, the larger is the perturbation. Thus, the APD S/N ratio is highest at peak flow. This difference must be compromised in the validation procedure.

Third, the APD can distinguish easily between resistance during inhalation and resistance during exhalation. The IOS unit used with the system described in this paper did not have this capability, so the IOS resistance reading was compared to the average of inhalation and exhalation resistances from the APD.

Lastly, the IOS machine requires a large power input in order to produce its signal in addition to a separate computer to operate, while the APD does not require any more power than to rotate the wheel and is completely self-contained. Thus, the APD can be small, portable, and compact, while the IOS machine is a large instrument. Whereas this difference does not directly influence the validation procedure, it does influence the physical layout of the components and the interface with the human volunteers.

## 2. Methods

### 2.1. Artificial Respiratory Model System

#### 2.1.1. System Configuration

The artificial respiratory model used for APD/IOS comparisons had three components analogous to a biological system: a sinusoidal flow source to model breathing, a compliance chamber to model lung compliance, and a controllable resistance to model respiratory resistance. 

First, a motorized syringe pump (no. 17050-3) purchased from VacuMed (Ventura, CA, USA) was selected to be used as the flow generator. The syringe pump was powered by a motor that generated a smooth, continuous sinusoidal flow. The magnitude and frequency of the flow was controlled by adjusting the stroke volume of the pump and the rotational frequency of the motor. In order to mimic human resting breathing, the stroke volume was set to 0.5 L for all experiments, and the motor frequency was set to 18 RPM to give a volumetric flow, $\dot{V}$, of 0.15 L · s^−1^. 

Second, a glass cylindrical container (height = 44.5 cm, inner diameter = 14 cm) was installed at the outlet of the piston pump to act as a compliance chamber. Because a positive displacement pump delivers nearly the same output regardless of downstream resistance, its effective internal resistance is uncontrollable and very high. If the fluid being delivered by the pump is incompressible, then the pump delivery would be determined solely by the pump and not by downstream resistance. Air, however, is compressible, so there is some effect of downstream resistance as air pressure in the piston cylinder increases or decreases. As a result, adding sufficient compliance to the flow pathway so that the extra downstream resistance causes the air to compress in the compliance chamber rather than to be forced through the resistance is one method to make a piston pump sensitive to downstream resistance changes, as required by the APD. 

Third, a series of resistances were installed following the compliance chamber. The resistance of the system was changed depending on the type and number of resistors installed. Two types of resistors were used to test two ranges of resistances. Fleisch no. 1 and no. 2 pneumotachographs (Phipps & Bird, Arlington, VA, USA) were used to provide a low range of resistance (0.12–0.31 kPa · L^−1^ · s or 1.2–3.2 cm H_2_O · L^−1^ · s), and Hans Rudolph standard flow resistors (Series 7100R2, Shawnee, KS, USA) were used to provide a high range of resistance (0.19–0.95 kPa · L^−1^ · s or 1.9–9.7 cm H_2_O · L^−1^ · s). Previous testing with the APD on people from ages 2 to 88 [24] has shown that this range incorporates the vast majority of expected resistance values.

**Figure 2:**
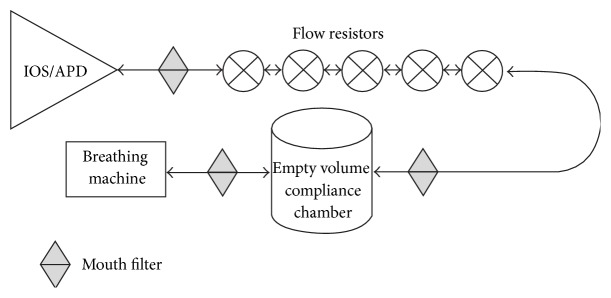
Schematic of the artificial respiratory model.

Tubing with rubber end connectors was used to connect the components. Antibacterial filters were also used as connectors between the components to ensure tight fits. Pneumotachographs were placed end-to-end and sealed with rubber connectors cut from mouth pieces in the Pulmonary Function Filter Kit purchased from AllianceTech Medical, Inc. (Granbury, TX). Hans Rudolph standard flow resistors were also placed end-to-end and connected with Hans Rudolph small connectors (Series 7023, 22 mm × 22 mm). A schematic of the complete system is illustrated in Figure 2.

#### 2.1.2. Calibration of the Resistors

The resistance of each individual resistor and each series combination of resistors was carefully measured from their steady-state flow-pressure relationships to provide a nominal value to compare with IOS and APD measurements. A steady flow of 0.16 L · s^−1^ was applied for these measurements. For pressure measurements, a Dwyer Model 40–1 (Michigan City, IN, USA) manometer was used, and to monitor flow, a Gilmont 40453 (Pelham, NH, USA) Flowmeter was used. Both the manometer and flowmeter were carefully calibrated. The four no. 2 pneumotachographs were labeled PT#2_1−4_, and the five Hans Rudolph standard flow resistors were labeled HR_1−5_. The no. 1 pneumotachograph was identified as PT#1. 

 The pressure/flow relationships of the pneumotachographs and Hans Rudolph Standard Flow Resistors were tested for linearity. The pressure drops across no. 1 and no. 2 pneumotachograph and Hans Rudolph standard flow resistor were recorded at various flows ranging from 0.05 to 0.70 L · s^−1^.

Plastic tubing was fit by compression onto the ends of each pneumotachograph, and each plastic tube was tapped to measure static pressure in close proximity to the pneumotachograph. The pressure taps on the pneumotachographs themselves were plugged with masking tape. The pressure drop was measured as the difference in pressures at the tubing pressure taps. This was done to obtain the entire pressure drop across the device and to account for end effects in the pneumotachs.

For the Hans Rudolph Standard Flow Resistors, Hans Rudolph small connectors were fit by compression onto the ends of each flow resistor. The pressure drop was measured across the pressure taps on these connectors.

To account for possible resistance differences due to resistor placement, total pressure drops were also obtained for series combinations of resistors. For the pneumotachographs, the pressure drop was recorded across each series combination as the number of pneumotachographs was increased incrementally up to four no. 2 pneumotachographs and one no. 1 pneumotachograph. For the Hans Rudolph Standard Flow Resistors, the number of resistors was increased incrementally from one to five resistors. 

#### 2.1.3. IOS and APD Measurements

A CareFusion (San Diego, CA, USA) MasterScreen IOS machine was used following the manufacturer specifications to measure the resistance of the validation system [25]. The IOS machine was attached to the output end of the system. Volume, temperature, and pressure calibrations were performed according to the manufacturer specifications. For each resistance configuration of the system, one IOS measurement of R5 was recorded and used for comparison. It was decided to use this R5 value as a good choice for comparison with APD measurements because R5 represents respiratory resistance over the entire respiratory system.

Unlike normal APD operating procedure, no previous APD flow or pressure calibrations were performed before usage. The perturbation frequency on the APD was set, as normally used, to 9.8 Hz, with two perturbations occurring with each rotation of the wheel. Consistent with the IOS procedure, five APD measurements were taken using the same APD unit. The reported inhalation and exhalation resistances were recorded as *R*_APD,in_ and *R*_APD,ex_, respectively. These values were averaged and reported as a single value, *R*_APD_.

### 2.2. Measurements on Human Subjects

Ten healthy nonasthmatic male and female volunteers between the ages of 18 and 30 years completed tests of respiratory resistance by the APD and the IOS machine. The perturbation frequency of the APD was set to 9.8 Hz. IOS measurements were made using a CareFusion (San Diego, CA, USA) MasterScreen IOS machine following the manufacturer specifications to measure resistance [25]. For both devices, an antibacterial filter from the Pulmonary Function Filter Kit purchased from AllianceTech Medical, Inc. (Granbury, TX, USA) was used as a mouthpiece for the test subjects. This protocol was approved by the University of Maryland Institutional Review Board.

Identical tests were performed on each subject using the APD and the IOS machines. Subjects performed four trials on the APD and one trial on the IOS machine. Inhalation and exhalation resistance values from the APD were averaged during each trial to give *R*_APD_. IOS measurements of R5 were recorded following completion of APD measurements to compare against *R*_APD_.

Respiratory resistance of the subjects was incrementally increased by adding various series combinations of Hans Rudolph Standard Flow Resistors (Series 7100R2) in between the test subject and the measuring device. The number of flow resistors was increased one at a time from one to four, giving a total range of 0.20–0.78 kPa · L^−1^ · s (2.00–8.00 cmH_2_O · L^−1^ · s) as indicated by the manufacturer, and measurements of respiratory resistance were taken at every increment. Resistors were connected by compression fitting using Hans Rudolph small connectors, and rubber adapters were used to connect resistor combinations to mouthpieces and devices.

## 3. Results

### 3.1. Calibration of the Resistors

*Individual Resistors*. The resistance of each resistor was calculated using the relationship between pressure and flow, $R=\Delta P/\dot{V}$. The resistance values were found to be consistent across all no. 2 pneumotachographs and across all Hans Rudolph Standard Flow Resistors. The resistance of the no. 1 pneumotachograph was found to be approximately as twice as that of a single no. 2 pneumotachograph, which is consistent with the manufacturer specifications.  Table 1 summarizes these findings.

Good linearity between pressure and flow was observed for the no. 1 and no. 2 pneumotachographs and the Hans Rudolph Standard Flow Resistors, for flow rates between 0.05 and 0.70 L · s^−1^. Dividing the pressure over the flow corresponded to the slope of the regression lines, giving a measure of the resistance value.  Figure 4 shows that the slopes of the regression lines (forced through zero) were within 5% agreement with the measured resistance values of each resistor listed in Table 1, with the exception of the no. 2 pneumotachograph, which was within 15% of the average (0.041 kPa · L^2−1^ · s compared to 0.048 kPa · L^−1^ · s, or 0.49 cm H_2_O · L^−1^ · s).

*Resistors in Series*. Resistors were coupled together in series in single unit increments for the pneumotachographs and the Hans Rudolph Standard Flow Resistors, and their resistances calculated from their pressure/flow relationships. Resistances of series combinations were approximately (but not exactly) equivalent to the algebraic sum of the individual resistors. The differences between the sum of individual resistor values and their combinations are likely due to small interfacing resistances incurred when connecting them together.  Table 2 summarizes these findings. 

**Figure 3:**
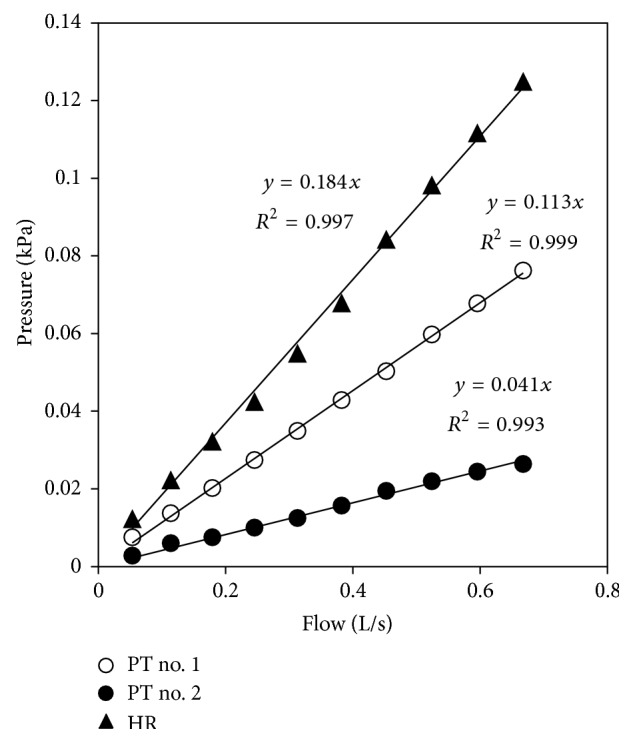
Pneumotachographs no. 1 and no. 2 (PT no. 1 and PT no. 2) and a Hans Rudolph standard flow resistor (HR) exhibit linear pressure and flow relationships at tested flows.

**Figure 4:**
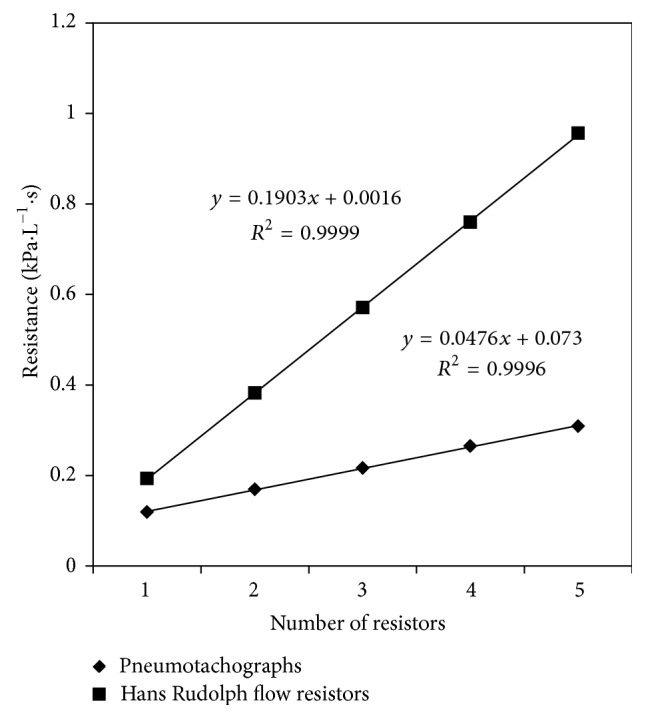
Increase in resistance in a series combination of resistors is linearly proportional to the number of resistors.

For both the series combination of pneumotachographs and the series combination of Hans Rudolph Standard Flow Resistors, the resistance was linearly proportional to the number of resistors in the system. Moreover, the proportionality for the series combination of both types of resistors was close to the averaged resistance of the individual resistors. This is represented by the slope of the linear regression shown in Figure 3. Specifically, for the series combination of pneumotachographs, the slope of the linear regression was equal to 0.047 kPa · L^−1^ · s per pneumotachograph, compared to 0.048 ± 0.003 kPa · L^−1^ · s (0.49 cm H_2_O · L^−1^ · s), the calibration average of the individual resistances of the no. 2 pneumotachographs. Similarly, for the series combination of Hans Rudolph Flow Resistors, the slope of the linear regression was equal to 0.190 kPa · L^−1^ · s per Hans Rudolph Flow Resistor, compared to 0.181 ± 0.002 kPa · L^−1^ · s (1.85 cm H_2_O · L^−1^ · s), the average calibration of the individual resistances of the Hans Rudolph Flow Resistors.

### 3.2. IOS and APD Measurements of the Physical System

**Figure 5:**
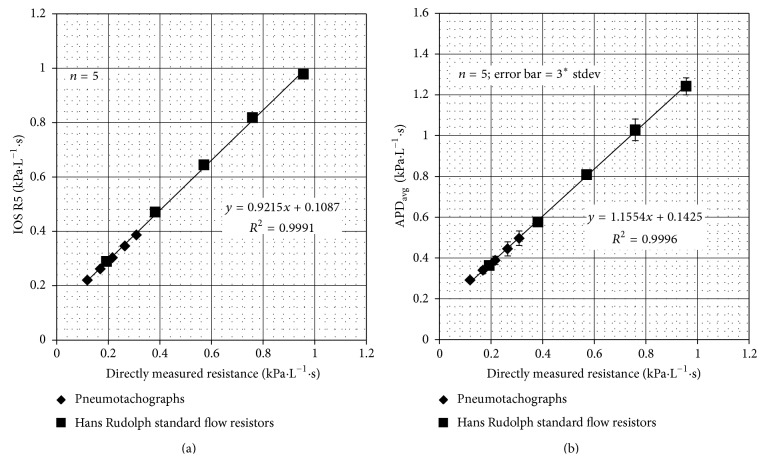
IOS R5 measurements of resistance of series combination of resistors slightly underestimated the directly measured resistance (a), whereas APD measurements of the same series combinations slightly overestimated the directly measured resistance (b). The offsets in both graphs are due to the unavoidable inherent resistance of the mechanical system without additional added resistances.

*IOS Measurements*. IOS measurements of R5 of the system were recorded for various series combinations of the pneumotachographs and the Hans Rudolph Standard Flow Resistors. In Figure 5(a), the resistance measured at 5 Hz, R5, was plotted against the directly measured resistance values (see Table 2), and a linear regression was performed. The data fit the regression curve exceptionally well (*R* ^2^ = 0.999). Moreover, the slope of the curve (0.921), an indication of how well IOS resistance measurements agree with directly measured resistances, suggested only slight underestimation by the IOS.

*APD Measurements*. Because the APD is capable of both inhalation and exhalation measurements of resistance, these measurements were recorded and averaged to produce the APD-measured resistance. In Figure 5(b), the APD-measured resistance values were plotted against the directly measured resistance values (see Table 2), and a linear regression was performed. Consistent with the IOS machine, the APD data also fit the regression curve exceptionally well (*R* ^2^ = 0.999). In contrast to the IOS machine, the slope of the curve (1.155) indicated slight overestimation by the uncalibrated APD.

**Figure 6:**
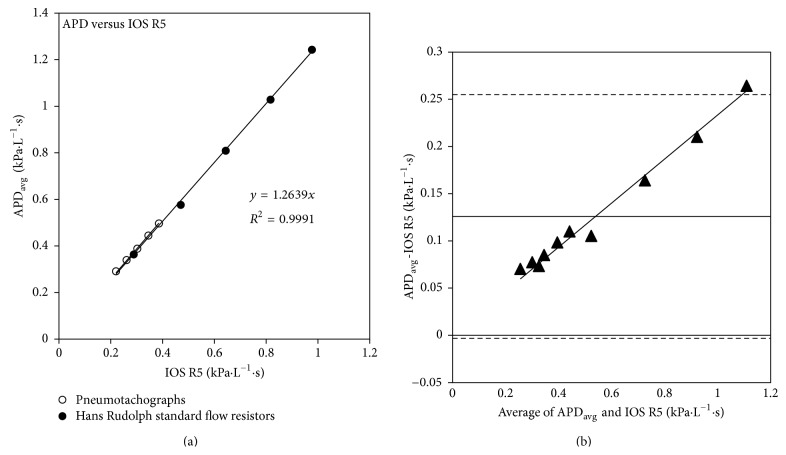
Strong linear correlation is observed between the IOS R5 measurements and the APD measurements (*R* ^2^ = 0.999) (a) as well as a strong proportional relation (*R* ^2^ = 0.985), shown in the Bland-Altman diagram (b). The dotted line represents ±1.96 standard deviation; the solid line represents the mean of the differences.

Despite the slight under and overestimations by the two devices, Figure 6 illustrates that a strong correlation exists between the two methods when one is compared against the other. A Bland-Altman diagram is typically used to compare two clinical measurements when the properties to be measured exhibit significant variation over their range such that correlation may not necessarily equate to agreement. In other words, two measures may be highly correlated yet exhibit substantial differences across their range of values. Here, the Bland-Altman diagram in Figure 6(b) illustrates that the difference between APD *R*_APD_ and IOS R5 depends highly on the magnitude of the resistance. This is indicative of a proportional relationship and conforms to the conclusions indicated by the data in Figure 5. 

### 3.3. APD and IOS Measurements of Human Subjects

**Figure 7:**
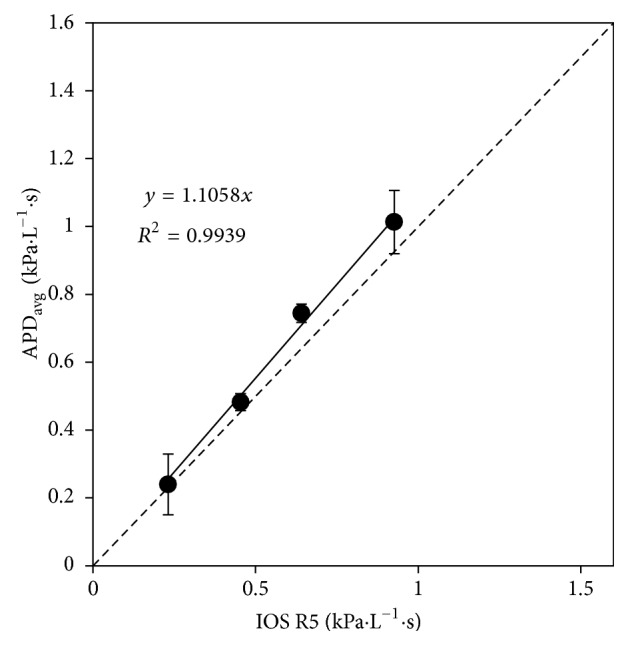
Uncalibrated APD-measured respiratory resistance at 9.8 Hz compared to IOS-measured respiratory resistance at 5 Hz for one typical subject, illustrating the high degree of linearity between the two devices. The linear regression was forced through zero. The dotted line indicates the line of identity. Points are averages of four trials. Error bars are three times the standard deviation.

Correlations between APD *R*_APD_ and IOS R5 were linear for all subjects breathing through several external resistors.  Figure 7 shows representative data from a typical subject, highlighting the high degree of linearity.  Table 3 lists the linear correlation factors as well as the goodness of fit, *R* ^2^. Linear regressions were forced through zero. The average correlation factor (1.208 ± 0.072) indicates slight overestimation by the APD compared with IOS. This is consistent with the previous results reported above that found a similar overestimation by the APD when measuring the resistance of the mechanical respiratory model. 

**Figure 8:**
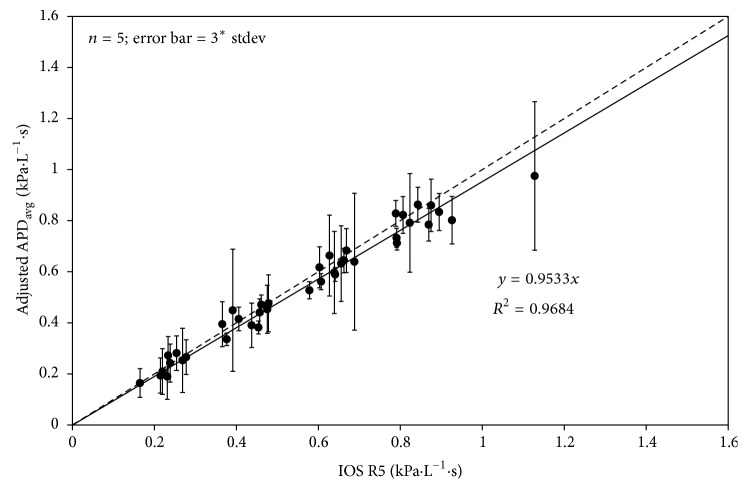
Average data of calibrated APD-measured respiratory resistances from the cohort of all ten test subjects are nearly identical to IOS-measured respiratory resistances. The linear regression was forced through zero. The dotted line indicates the line of identity. Points are averages of four trials. Error bars are three times the standard deviation.

The APD was calibrated to the IOS using the calibration factor (1/1.263) obtained from the comparison made on the mechanical system, and the data from the ten subjects were plotted in Figure 8. Both the calibrated APD and IOS gave nearly identical resistance measurements, with less than 5% difference between the two. This difference is less than the natural variation inherent in the respiratory system [26]. 

## 4. Discussion

Here, we presented data that illustrate that the APD is capable of making airflow resistance measurements comparable to those made by the commercially-available IOS instrument. This was shown on a nonbiological, mechanical respiratory system as well as on human subjects. The APD produced measurements as consistently as those made by IOS. 

The APD used in these experiments was not calibrated for prior pressure or flow accuracy. Normally, it would be expected that each individual APD would require calibration either against the IOS system or against the calibrated resistor combination to assure consistency among devices. This experiment has provided the means to do so. In the past, different APD devices gave slightly different respiratory resistance measurements due to variations in individual component parts. This no longer needs to be the case. The ratio of APD measurement values to IOS measurement values provides a calibration factor that can be used to calibrate APD average resistances. The fact that this factor is nearly the same for human subjects as it is for a mechanical system means that the highly reproducible and reliable mechanical system can be used for this purpose. The coefficient of variation of the correlation factors for all human subjects, an indicator of how random the amount of overestimation is, was 5.92%, suggesting that measurements on human beings are probably not the best to use for calibration purposes. A study by Johnson et al. [26] showed that there is a substantial variation in the average respiratory measurements that comes from the human respiratory system and not from the APD. Using the calibration factor obtained from the measurements on the mechanical system gave agreement within 5% of human subject respiratory resistances obtained from both devices. Considering that resistance measurements on humans can vary by much more than this, about 10%, the agreement by the calibrated APD with the IOS is certainly acceptable.

Both APD and IOS measurements correlated well with directly measured resistances of the series combinations, but a slight underestimation by the IOS machine and a slight overestimation by the APD were observed. For the IOS machine, this error may be due to the compressive nature of air in the compliance chamber. Although the inclusion of a compliance component also makes the physical system similar to a human respiratory system, the intent of the compliance chamber was to provide a volume in which the downstream resistance would force air to compress during every stroke of the piston pump. In this way, airflow becomes sensitive to downstream resistance and not just the cycling of the pump, allowing for the measurements of the resistance with the APD. However, since the impulse that the IOS machine emits is itself a pressure wave, it may become attenuated by the air within the compliance chamber, resulting in a lower measurement of R5. In fact, when no resistors were attached, the IOS machine gave no reading at all, presumably because the entire impulse was being attenuated (data not shown). 

**Figure 9:**
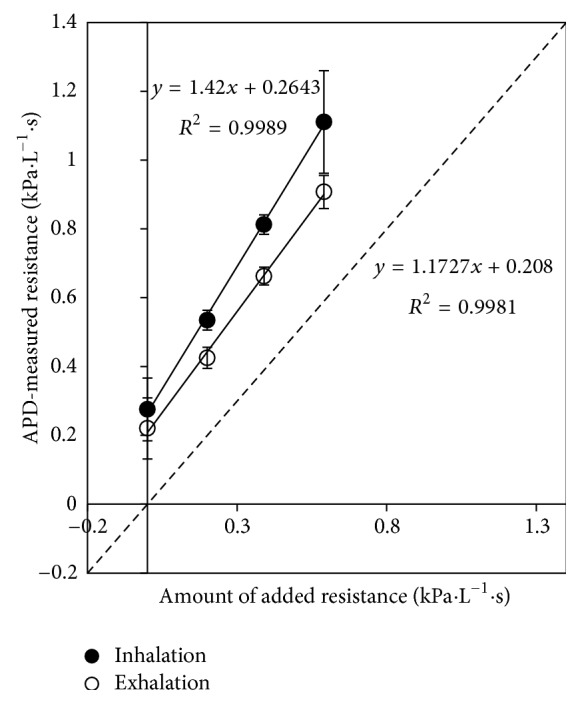
APD-measured respiratory resistance indicates higher respiratory resistance in the inhalation direction compared to the exhalation direction for one particular subject. The APD used for this measurement was uncalibrated. The dotted line indicates the line of identity. Points are averages of four trials. Error bars are three times the standard deviation. The offsets for the subject data are respiratory resistances that are located inside the respiratory system of the subject.

 Additionally, differences between inhalation respiratory resistance and exhalation respiratory resistance can give insight into diagnosing certain respiratory pathologies. One powerful feature of the APD is its ability to resolve these directions. For example, Figure 9 illustrates a sample test subject who appears to exhibit higher respiratory resistance in the inhalation direction than the exhalation direction. It would be expected that exhalation resistance should be higher than inhalation resistance because of the distensible airways and high external pressures during exhalation [27]. This subject may have a respiratory problem, or the difference may be due to the fact that the APD used in these tests was uncalibrated.

**Figure 10:**
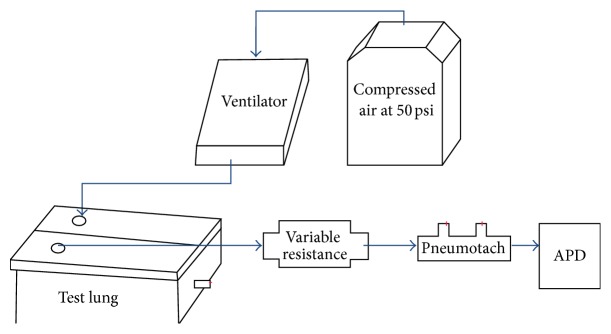
Airflow through the ventilator test lung system. Arrows indicate flexible plastic tubing connections.

Some might suggest alternate means to fabricate a mechanical system to be used for calibration purposes. A previous attempt at APD validation was based upon a ventilator test lung system (Dual Adult Training and Test Lung, Michigan Instruments, Grand Rapids, MI, USA) that relied on the compliance of the test lung to soften the flow source. One of the two parallel test lungs was supplied through a ventilator with compressed air at 50 psi and set to 10 BPM with a 3 second inspiration time to mimic human resting breathing. This raised the second lung mechanically coupled to the first. The flow from the second test lung was subsequently fed into the APD through interchangeable orifices (Pneuflo Rp5 and Rp20, Michigan Instruments) used as resistances.  Figure 10 shows a diagram of the airflow path through the system. 

The problems that were encountered with this system were the inconsistency between trials (probably due to flow rate dependence of the orifice resistances) and APD resistance values that differed considerably from the resistance values supplied by the manufacturer. By contrast, the current system diagrammed in Figure 2 gave very consistent measurements. 

 Based on the results given in this paper, we can conclude that the APD compares well with the IOS method, and it can be an accurate method to assess respiratory resistance in patients.

## Figures and Tables

**Table 1:** Measured resistance values of each resistor.

Pneumotachographs	Resistance (kPa·L^−1^·s)	Hans Rudolph standard flow resistors	Resistance (kPa·L^−1^·s)
PT no. 1	0.109	HR_1_	0.182
PT no. 2_1_	0.044	HR_2_	0.179
PT no. 2_2_	0.051	HR_3_	0.182
PT no. 2_3_	0.051	HR_4_	0.179
PT no. 2_4_	0.049	HR_5_	0.182

**Table 2:** Measured resistance values of series combinations of resistors.

Pneumotachographs	Resistance (kPa·L^−1^·s)	Hans Rudolph standard flow resistors	Resistance (kPa·L^−1^·s)
PT no. 1	0.119	HR_1_	0.194
PT no. 1 + PT no. 2_1_	0.169	HR_1-2_	0.382
PT no. 1 + PT no. 2_1-2_	0.217	HR_1–3_	0.571
PT no. 1 + PT no. 2_1–3_	0.265	HR_1–4_	0.759
PT no. 1 + PT no. 2_1–4_	0.309	HR_1–5_	0.956

**Table 3:** All subjects showed high linearity between APD-measured respiratory resistance and IOS-measured respiratory resistance, as indicated by strong goodness of fit coefficients. In addition, all subjects also showed slight overestimation by the APD compared with IOS R5.

	Correlation factor	Goodness of fit (*R* ^2^)
Subject no. 1	1.306	0.997
Subject no. 2	1.198	0.997
Subject no. 3	1.105	0.993
Subject no. 4	1.265	0.993
Subject no. 5	1.160	0.912
Subject no. 6	1.142	0.998
Subject no. 7	1.149	0.998
Subject no. 8	1.312	0.993
Subject no. 9	1.247	0.992
Subject no. 10	1.197	0.977
